# Duloxetine-Induced Neural Cell Death and Promoted Neurite Outgrowth in N2a Cells

**DOI:** 10.1007/s12640-020-00216-x

**Published:** 2020-05-16

**Authors:** Wanli Gao, Rui Chen, Nan Xie, Daolin Tang, Borong Zhou, Ding Wang

**Affiliations:** 1 Key Laboratory for Major Obstetric Diseases of Guangdong Province, Key Laboratory of Reproduction and Genetics of Guangdong Higher Education Institutes, Center for DAMP Biology, The Third Affiliated Hospital of Guangzhou Medical University, Guangzhou, 510510 People’s Republic of China; 2 Department of Neurology, The Third Affiliated Hospital of Guangzhou Medical University, Guangzhou, 510510 People’s Republic of China; 3 Department of Reproductive, The Third Affiliated Hospital of Guangzhou Medical University, Guangzhou, 510510 People’s Republic of China; 4 Department of Oral Pathology, Guanghua School of Stomatology, Research Institute of Stomatology, Guangdong Province Key Laboratory of Stomatology, Sun Yat-sen University, Guangzhou, 510055 Guangdong People’s Republic of China; 5 Department of Surgery, UT Southwestern Medical Center, Dallas, TX 75390 USA; 6 Experimental Department of Institute of Gynecology and Obstetrics, The Third Affiliated Hospital of Guangzhou Medical University, Guangzhou, 510510 People’s Republic of China

**Keywords:** Duloxetine, N2a cells, Neural cells, Cell death, Neurite outgrowth

## Abstract

Duloxetine is a clinical drug that is primarily used for treatment of depression and pain, but it has side effects of addiction and tolerance. Cytochrome P450 (CYP) is its metabolic enzyme, and the drug’s biofunction results from its neuro-protective effect in animal and cell models. We aimed to investigate the duloxetine-induced neural cytotoxicity effect and its performance in an N2a cell neurite outgrowth model. Cell death was assessed as cell viability using a Cell Count Kit-8 and further evaluated using bright-field images, propidium iodide (PI) and annexin V staining, colony-formation analysis, TUNEL staining of the cells, and biochemical testing. N2a cells were committed to differentiation by serum withdrawal and RA induction, and the neurite outgrowth was evaluated as the number of differentiated cells, longest neurite length, and average neurite length. Cell cycle analysis, PI and annexin V staining, mRNA expression, and biochemical testing were used to evaluate the drug effects on differentiation. The induction of neural cell death by duloxetine was not affected by classic cell death inhibitors but was promoted by the CYP inducer rifampicin. N2a cell neurite outgrowth was promoted by duloxetine via reduction of the CYP2D6 and MDA levels and induction of Bdnf protein levels. Duloxetine induces neural cell death through effects on CYP and promotes N2a cell neurite outgrowth by regulating CYP, Bdnf protein, and the intracellular lipid peroxidation level.

## Introduction

Duloxetine is a psychoactive antidepressant drug that is licensed for treatment of depression, anxiety, fibromyalgia, neuropathic pain, and incontinence. In clinical trials, duloxetine showed efficacy in improving neurologic symptoms and pain relief. Furthermore, duloxetine exhibited excellent efficacy in treating pain in diabetic peripheral neuropathy and fibromyalgia compared with other antidepressant drugs (Lunn et al. 2009; Lunn et al. 2014). However, there have also been unfavorable views of duloxetine, in which it was considered to be harmful (Spence 2014). First, duloxetine carries a potential addiction risk, which is a common problem with all psychoactive drugs. Furthermore, for the other indication, namely, depression, the efficacy of duloxetine did not show a significant advantage in the treatment of the acute-phase of major depression compared with other antidepressive agents and was even worse in terms of acceptability and tolerability (Cipriani et al. 2012). Duloxetine has been used clinically for several decades and is widely administered today, but studies of the cell biology of its pharmacology and cytotoxicity have been limited.

The biological effects of duloxetine with respect to its pharmacology and pharmacokinetics have been studied. Its psychiatric effects target the central nervous system (CNS), and it is catabolized in liver. Like some other agents (known as a serotonin–norepinephrine reuptake inhibitors, SNRIs), duloxetine is a potent inhibitor of serotonin (SER, 5-HT), and norepinephrine reuptake (NOR) is a weak inhibitor of dopamine transporters, and has low binding affinity for other neurotransmitter receptors (Carter and McCormack 2009), which results in high concentrations of SER and NOR in the CNS in the treatment of psychiatric illness. The biotransformation of duloxetine is the result of cytochrome P450 (CYP) enzymes. In an in vitro study, CYP2D6 level was inhibited by a very low concentration of duloxetine; in vivo, a CYP1A2 inhibitor caused a significant increase in the C_max_ (maximum plasma concentration) of duloxetine, this indicated that both CYP2D6 and CYP1A2 participate in duloxetine metabolism (Knadler et al. 2011). The tolerability of duloxetine, which is limited by factors such as nausea, dry mouth, headache, constipation, dizziness, and fatigue, is probably associated with its influence on CYP. In the literature, we did not find any evidence for duloxetine-induced neurotransmitter and CYP changes in neural cells.

Identification of duloxetine’s toxicity and neural biological function would improve the understanding of the drug’s pharmacology and interpretation of its side effects, but few of the physiologically relevant functions of duloxetine have been recognized. In vivo, most of the literature has indicated that duloxetine had a neuro-protective effect in animal models. The mechanisms involved included upregulation of brain-derived neurotrophic factor (BDNF) (Mannari et al. 2008), an anti-inflammatory effect (Choi et al. 2015) and suppression of the glial functions (Tawfik et al. 2018), but it was stated that duloxetine did not result in any physiological effect on neurogenesis (Marlatt et al. 2010). In vitro, duloxetine protected cultured neurons (Demirdas et al. 2017) and neural cells (Akpinar et al. 2014) against stress induced by oxidative stress, apoptosis, and Ca^2+^ entry. However, there were no data regarding duloxetine’s neural cytotoxicity and its biofunction on the induction of neurite outgrowth.

In this study, we investigated duloxetine’s neural cytotoxicity and the associated cell death events using concentrations ranging from the in vivo biological levels to the median lethal concentrations in two mouse neural cell lines, neural progenitor cells (C17.2 cells), and neuroblastoma cells (N2a cells). Furthermore, we evaluated the biofunction of duloxetine in N2a cells’ neurite outgrowth and found out that duloxetine promoted neurite outgrowth of N2a cells by decreasing the intracellular CYP2D6 and MDA levels, and increasing the Bdnf protein levels of intracellular and extracellular. Collectively, these findings revealed that duloxetine is a potential anti-neuroblastoma cell agent and a promoter of neural network reconstruction.

## Methods

### Reagents

Duloxetine (duloxetine hydrochloride, Cat#HT-B0161A), trolox (Cat#HY-101445), folic acid (Cat#HY-16637), rifampicin (Cat#HY-B0272), retinoic acid (RA) (Cat#HY-14649), rapamycin (Cat#HY-10219), chloroquine (Cat#HY-17589), and necrostatin-1 (Cat#HY-15760) were purchased from MCE (MedChemExpress, Shanghai, China). Z-VAD (Cat#S7023) and ferrostatin-1 (Cat#S7243) were purchased from Selleck Chemicals (Houston, TX, USA).

### Cell Culture

Mouse neuroblastoma cells (N2a cells) were purchased from ATCC (American Type Culture Collection), and mouse neural progenitor cells (C17.2 cells) were purchased from ECACC (European Collection of Cell Culture). N2a cells were cultured in Dulbecco’s Modified Eagle’s Medium (DMEM) supplemented with 10% fetal bovine serum (FBS) (Cat#10099141, Gibco, Thermo Fisher Scientific, California, USA), 100 U/ml penicillin and 100 μg/ml streptomycin at 37 °C in a humidified atmosphere of 5% CO_2_. C17.2 cells were cultured as described for the N2a cell protocol except that 5% horse serum was added to the medium (Cat#26050070, Gibco). The two cell lines were grown to 80% confluence, and then passaged by trypsin at a ratio of 1:4. The cell morphology of duloxetine-induced cytotoxicity and neurite outgrowth was recorded using a living-cell imaging system: a Leica MC170HD microsystem (Leica Microsystems, Wetzlar, Germany).

### Cell Viability Assay

Cells were seeded in 96-well plates at a density of 2 × 10^5^ cells per well, grown for 24 h, and then treated with the drugs according to time-dependence or dose-dependence protocols. Each treatment was conducted in triplicate. After the drug treatments, the cell viability was assayed using a Cell Counting Kit-8 (CCK-8) (Cat#CK04-3000T, Dojindo Laboratories, Japan) according to the manufacturer’s instructions. CCK-8 uses the sensitive colorimetric WST-8 assay to determine the number of viable cells. WST-8 is a highly water-soluble tetrazolium salt, with the chemical designation of 2-(2-methoxy-4-nitrophenyl)-3-(4-nitrophenyl)-5-(2,4-disulfophenyl)-2H-tetrazolium, monosodium salt.

### Colony-Forming Assay

N2a cells were treated with the various indicated concentrations of duloxetine for 24 h. Triplicate wells of 6-well plates containing 1 × 10^3^ cells were treated with various concentrations of duloxetine and maintained for another 21 days. The colonies were fixed with methanol, stained with a 0.1% crystal violet solution (Cat#G1064, Solarbio, Beijing, China) in 1 h at room temperature, and counted. The colony formation assay was repeated three times.

### N2a Cell Differentiation

N2a cells were differentiated by a protocol that involved RA addition and serum withdrawal. The differentiation medium was DMEM supplemented with 20 μM RA, 100 U/ml penicillin and 100 μg/ml streptomycin. Neurites were identified as cell processes greater than two cell body diameters in length. Differentiated cells were defined as those bearing neurites. The percentage was statistically analyzed by counting 180 cells in six randomly chosen fields per well. The neurite length was defined as the distance from the cell body to the tips of the neurites. The length of the longest neurite was measured in at least 50 cells in five randomly chosen fields using the ImageJ software. To evaluate the cell toxicity, N2a cells that were committed to differentiation with RA were treated with the addition of 12.5 μM duloxetine or 12.5 μM duloxetine plus 10 μM rifampicin for 24 h. Statistical comparisons of the cell morphology were conducted between control, RA, RA + duloxetine and RA + duloxetine + rifampicin groups after a 24-h treatment (*n* = 3). The cell viability and cell morphology were recorded each day during the full differentiation period (*n* = 3). Furthermore, the events associated with cell cycle and cell death were analyzed at various time points in the control and RA groups to permit interpretation of the key changes during the N2a cell differentiation. Statistical analyses of cell death (*n* = 4), cell cycle (*n* = 4) and biochemical changes (*n* = 3) were conducted in the control, RA, RA + duloxetine groups after the 24-h treatment.

### Lactate Dehydrogenase Assay

Lactate dehydrogenase (LDH) is an intracellular enzyme that is released to the supernatant during cell death. The LDH release into the incubation medium after cell membrane damage was measured using an LDH diagnostic kit (Cat#C0016, Beyotime, Shanghai, China) according to manufacturer’s instructions. There were three repeats of each group for statistical analysis.

### Lipid Peroxidation Assay

The lipid peroxidation level was determined by measuring the concentration of malondialdehyde (MDA), which is the end product of lipid peroxidation and reacts with TBA to form a fluorescence adduct. The total MDA quantities were determined using a Lipid Peroxidation MDA Assay Kit (Cat#S0131, Beyotime) according to manufacturer’s instructions. The total protein content was determined using the Pierce BCA Protein Assay Kit (Cat#23227, Thermo Fisher Scientific). The MDA level for each group was the total MDA divided by the total protein. There were three repeats of each group for statistical analysis.

### Cell Death Assay

Cell death was determined by flow cytometric analysis of annexin V- and propidium iodide (PI)-positive cells and photography of TUNEL (TdT-mediated dUTP Nick-End Labeling) positive cells. For flow cytometry, the cell samples were trypsinized to single cells and stained using an annexin V-FITC apoptosis-detection kit (Cat#C1062M, Beyotime) according to manufacturer’s instructions. The populations of annexin V- and PI-positive cells were measured using flow cytometry (AttuneTM NxT Acoustic Focusing Cytometer, A24863, Thermo Fisher Scientific). For photography, the cells were fixed with 4% paraformaldehyde and stained using a TUNEL-FITC kit (Cat#11684817910, Roche, Basel, Switzerland) according to manufacturer’s instructions. DAPI staining was used to determine the total cell number. For statistical analysis, the positive ratios were determined in three visual fields using fluorescence microscope photography (TE2000, Nikon, Tokyo, Japan).

### Cell Cycle Analysis

The populations of cells in the phases of the cell cycle were determined by DNA content as indicated by staining with PI. The cell samples were fixed with 70% cold ethanol at 4 °C overnight. PI staining was conducted using a cell cycle and apoptosis analysis kit (Cat#C1052, Beyotime) according to manufacturer’s instructions. Flow cytometric analysis of the fluorescence intensity was used to evaluate the various phases of the cell cycle.

### Enzyme-Linked Immunosorbent Assay

Enzyme-linked immunosorbent assays (ELISA) were used to determine the serotonin (Cat#BAE-5900, Rocky Mountain Diagnostics, Colorado Springs, CO, USA) norepinephrine (Cat#BAE-5200, Rocky mountain diagnostics), Bdnf (Cat#EK2127, Multi Sciences, Shanghai, China), cytochrome P450 (CYP) 1A2 (Cat#xyD294Ra, IBL-America, Minneapolis, MN, USA), and CYP2D6 (Cat#xyD302Ra, IBL-America) protein levels. For the cell culture supernatant assays, samples of the medium were collected and immediately frozen at − 80 °C. For the assays of the intracellular levels, the cells were collected and lysed using liquid nitrogen, and the cell lysate dilutions were determined on the basis of the total protein assayed using the BCA kit. The concentrations were calculated according to standard curves prepared on the same ELISA plates according to the manufacturer’s instructions. There were three repeats of each group for statistical analysis.

### Quantitative Real-time Polymerase Chain Reaction Analysis

Total RNA was extracted from different drug treatment groups using TRIzol™ (Invitrogen, Cat# 12183555, Carlsbad, CA, USA). A total of 1 μg RNA was used to synthesize cDNA by PrimeScript™RT Reagent Kit (Takara Biotechnology, Cat# RR047A, Kyoto, Japan). SYBR® Premix Ex Taq™ II (Takara Biotechnology, Cat# RR820A) was used for quantitative PCR, which was conducted on a StepOneTM Real-time PCR System (Thermo Fisher Scientific, Cat#4376373) and analyzed using the ViiA7™ System software (Thermo Fisher Scientific). The mRNA levels were calculated using the ddCt method relative to the expression of 18s RNA, which was a housekeeping gene, (as control). The mouse specific primers for RT-PCR were listed as follows: Bdnf forword, 5′- GGCTGACACTTTTGAGCACGTC-3′, Bdnf reverse, 5′- CTCCAAAGGCACTTGACTGCTG-3′, 18S forword, 5′-GCCATGCATGTCTAAGTACGC-3′, and 18S reverse, 5′-TCTGATAAATGCACGCATCC-3′.

### Statistical Analysis

The means ± SD were used to express quantitative results, and scatter plots were used to express proportional results. The comparisons of two groups were statistically analyzed using unpaired Student’s *t* tests. ANOVA (analysis of variance) was used for comparisons among multiple groups, such as those for time- or dose-dependent changes. *P* values of < 0.05 were considered statistically significant for all.

## Results

### Duloxetine-Induced Neural Cell Death

**Fig. 1:**
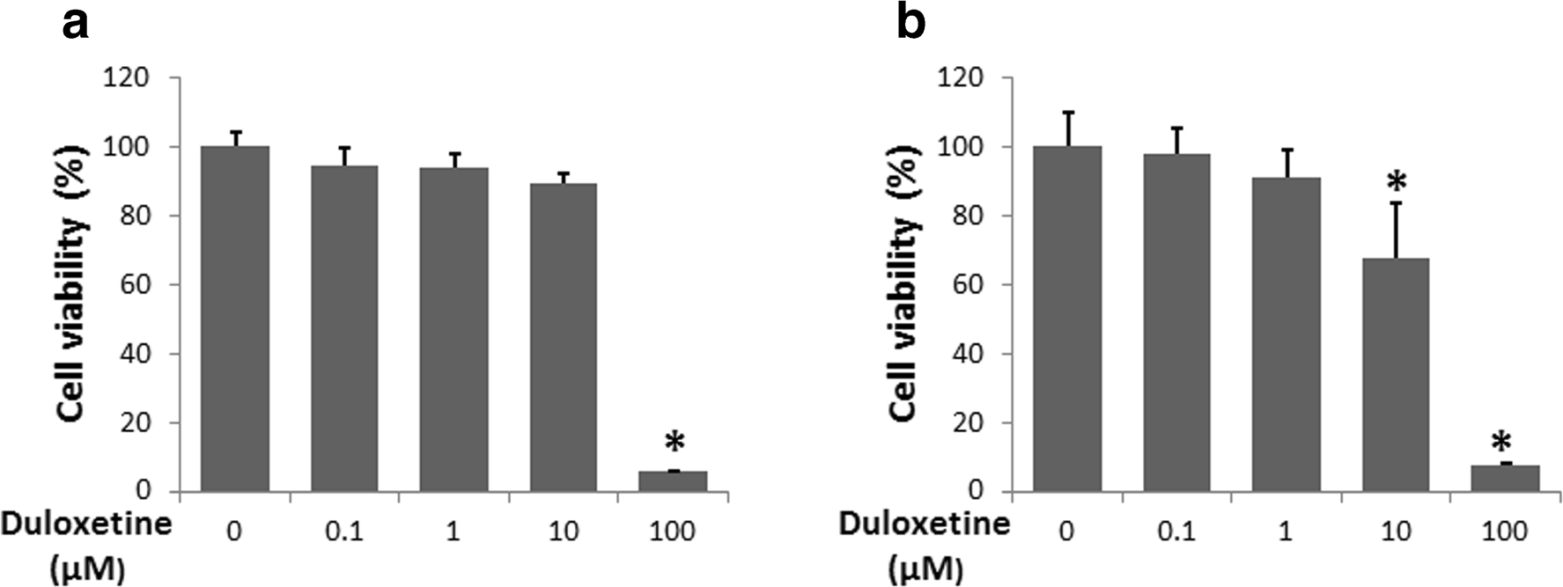
Duloxetine-induced neural cell death was not associated with serotonin and norepinephrine. Cell viability is indicated for N2a cells (**a**) and C17.2 cells (**b**) treated with 0.1 to 100 μM duloxetine for 24 h. (*n* = 3, *P < 0.05 versus control group)

Neural cells were treated for 24 h (hours) with concentrations of duloxetine ranging from a minimum of 0.1 μM to a maximum of 100 μM. There were no significant changes in either cell type in the 0.1 μM and 1 μM groups. For the N2a cells, there was a significant decrease in the 100 μM group compared with the control group (*P* < 0.01) (Fig. 1a). For C17.2 cells, the cell viability was significantly decreased in the 10 μM and 100 μM groups (Fig. 1b). Because the main biofunction of duloxetine is serotonin–norepinephrine reuptake inhibition, we assessed the changes in the concentrations of serotonin and norepinephrine in N2a cells. There were no significant changes in serotonin and norepinephrine, neither the extracellular nor intracellular levels.

**Fig. 2:**
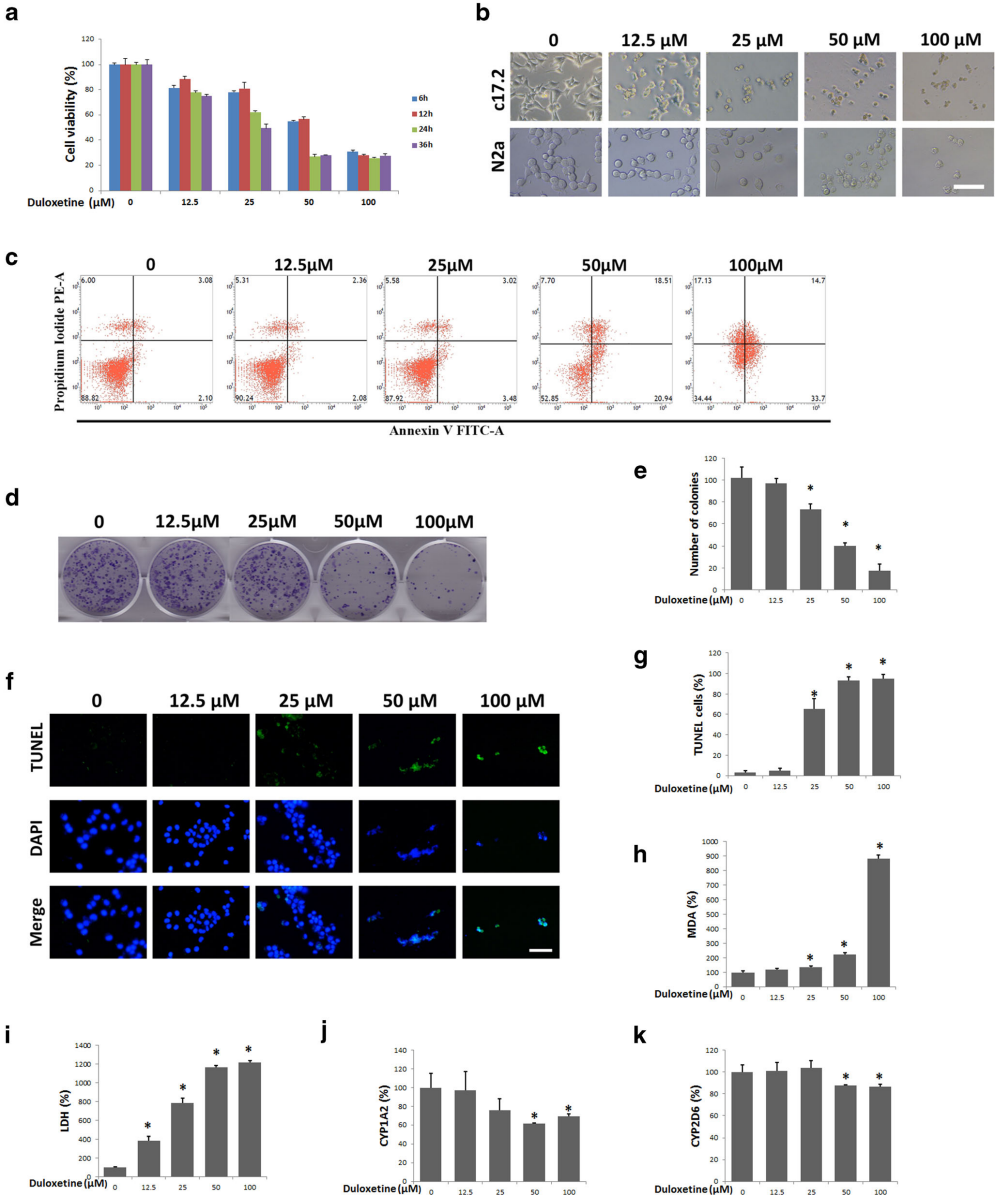
Duloxetine-induced neural cell death reduced CYP level. Duloxetine induced changes in the N2a cell viability in dose-dependent and time-dependent manners (**a**). Bright-field images are shown for N2a cells and C17.2 cells that were treated with a range of concentrations of duloxetine for 24 h (**b**). The following cell death events were assayed in N2a cells that were treated with various concentrations of duloxetine for 24 h. Annexin V- and PI-positive cells (**c**), colony-formation ability (**d** and **e**), TUNEL-positive cells (**f** and **g**), MDA (**h**), LDH (**i**), CYP1A2 (**j**), and CYP2D6 (**k**) protein levels were assayed and analyzed statistically. (*n* = 3, *P < 0.05 versus control group)

Because treatment with 1 μM duloxetine for 24 h did not exhibit any cell-killing effect in the N2a cells, we tested four concentrations (12.5 μM, 25 μM, 50 μM, and 100 μM) and four time points (6 h, 12 h, 24 h, and 36 h) to determine the dose-dependent and time-dependent characteristics of duloxetine toxicity. Duloxetine caused significant time- and dose-dependent changes in N2a cell viability (Fig. 2a). Then, we evaluated the dose-dependence of the duloxetine-induced cell death at 24 h. In bright field microscopy, the cell morphology of the N2a and C17.2 cells was typically changed, including less distinct cell boundaries and cell shrinkage (Fig. 2b). N2a and C17.2 cells exhibited similar trends of dose-dependent duloxetine-induced cell death. Because the N2a cells were more tolerant of duloxetine toxicity, we evaluated the following cell death events in N2a cells. Duloxetine-induced changes in the N2a cell populations as follows: an increase in the annexin V- and PI-positive cells (Fig. 2c); a decrease in the colony-formation ability (Fig. 2d); and an increase in the TUNEL-positive cells (Fig. 2f), all of which were significant in the 25 μM, 50 μM, and 100 μM groups (Fig. 2 e and g). Duloxetine-induced biochemical changes in the N2a cells included increased levels of MDA in the cell lysates (Fig. 2h) and LDH (Fig. 2i) in the cell culture supernatants and decreases in the protein levels of CYP1A2 (Fig. 2j) and CYP2D6 (Fig. 2k) in the cell culture supernatants in a dose-dependent manner.

### Rifampicin Promote Duloxetine-Induced N2a Cell Death

**Fig. 3:**
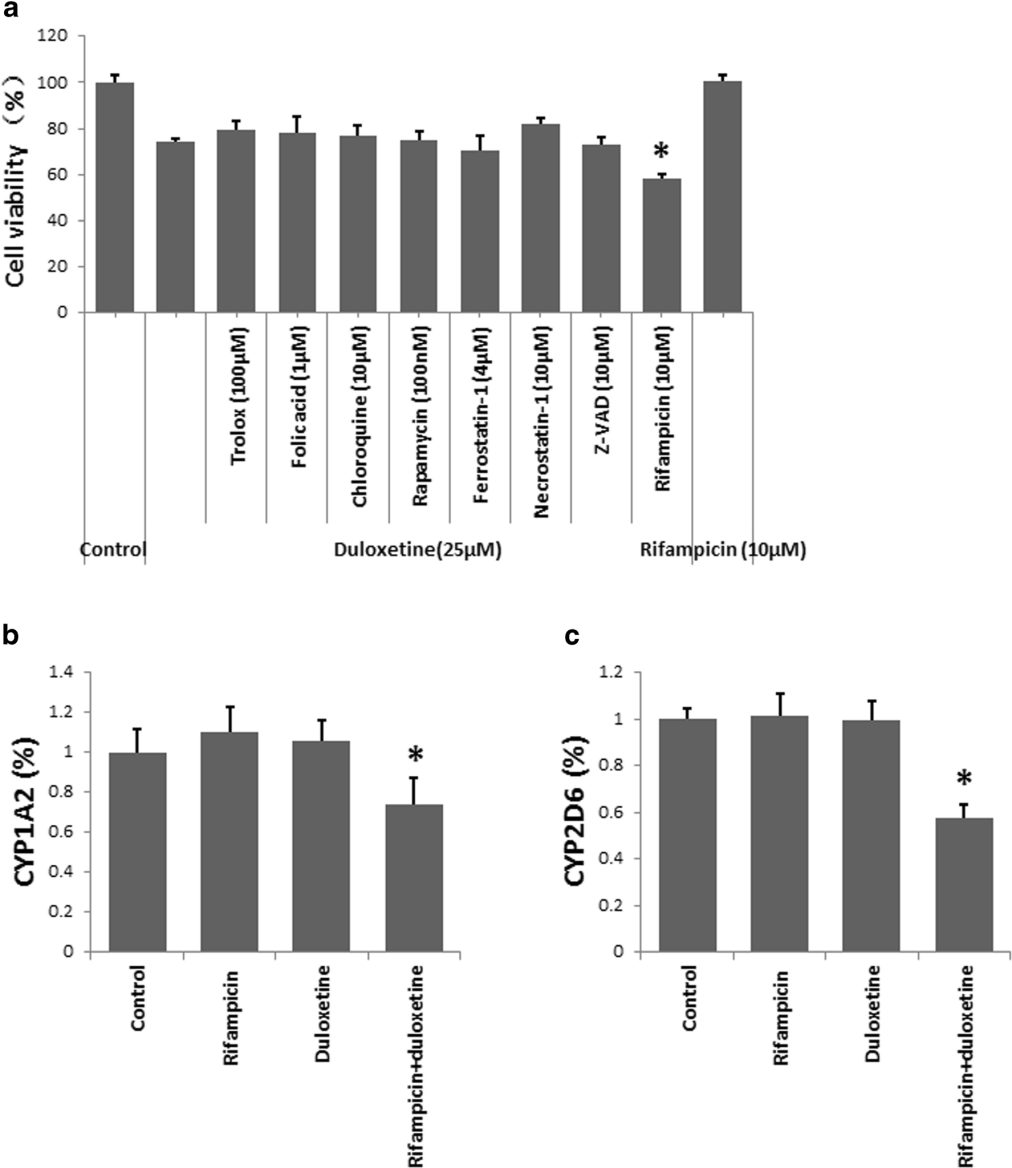
Duloxetine-induced N2a cell death was promoted by rifampicin. The viability of N2a cells after co-treatment of duloxetine and classic cell death inhibitors for 24 h is shown (**a**). N2a cells were treated by rifampicin (10 μM), duloxetine (25 μM), and the combination of these agents for 24 h, after which CYP1A2 (**b**) and CYP2D6 (**c**) protein levels were analyzed statistically. (*n* = 3, *P < 0.05 versus duloxetine group)

With the exception of rifampicin, the inhibitors did not affect the death of the N2a cells induced by 25 μM duloxetine. Although 10 μM rifampicin did not affect N2a cell viability independently, this inhibitor significantly promoted the N2a cell death induced by 25 μM duloxetine (Fig. 3a). Furthermore, we examined the change in the CYP level in N2a cells that were treated with 10 μM rifampicin and 25 μM duloxetine. At these doses, rifampicin and duloxetine did not affect CYP protein levels individually, but the combination significantly reduced the CYP1A2 (Fig. 3b) and CYP2D6 levels.

### Duloxetine Promotes Outgrowth of Neurites in N2a Cells

**Fig. 4:**
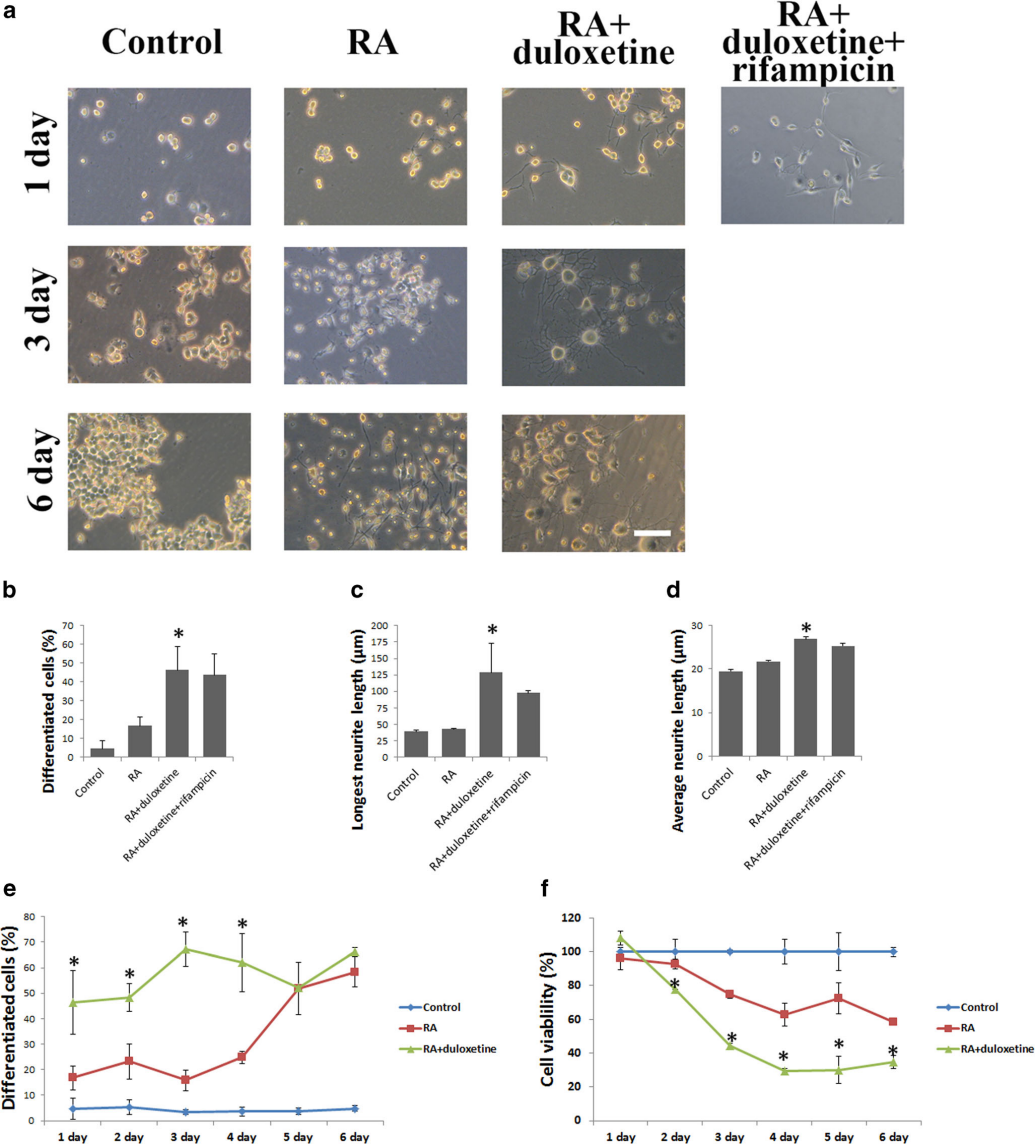
Neurite outgrowth of N2a cells was promoted by duloxetine. The statistical analyses of the cell morphology (**a**) and neurite outgrowth (**b** for percentage of differentiated cells, **c** for longest neurite length, and **d** for average neurite length) are indicated for the N2a cells that were treated with duloxetine (12.5 μM) or the combination of duloxetine and rifampicin (10 μM) for 24 h. The further differentiation of N2a cells was evaluated without duloxetine: the cell morphology (**a**), percentage of differentiated cells (**e**) and cell viability (**f**) were recorded and analyzed statistically. (*n* = 3, *P < 0.05 versus RA group)

Two differentiation groups were treated with 12.5 μM duloxetine or 12.5 μM duloxetine with 10 μM rifampicin during the first day of differentiation (Fig. 4a). There were significant increases in the numbers of differentiated cells (Fig. 4b), longest neurite length (Fig. 4c), and average neurite length (Fig. 4d) in the duloxetine group compared with the RA group, but there was no significant difference between the duloxetine + rifampicin group and the duloxetine group. To examine the duloxetine toxicity, the subsequent differentiation of the duloxetine group was observed for 6 days after withdrawal of the duloxetine (Fig. 4a). We monitored the differentiation ratio and cell viability of the N2a cells each day during these 6 days. Duloxetine significantly promoted neurite outgrowth in the N2a cells in first 4 days following the 24-h treatment compared with control RA differentiation protocol, but did not affect the final differentiated cell rate (Fig. 4e). The cell viability of the N2a cells was not attenuated after the 24-h duloxetine treatment, but this treatment led to a significant reduction in the last 5 days (Fig. 4f).

### Duloxetine Promoted Neurite Outgrowth of N2a Cells by Decreasing the Intracellular CYP2D6 and MDA Level, and Increasing Bdnf Levels

Flow cytometric analysis and biochemical assays were used to infer the mechanism for the duloxetine-induced neurite outgrowth in the N2a cells.

**Fig. 5:**
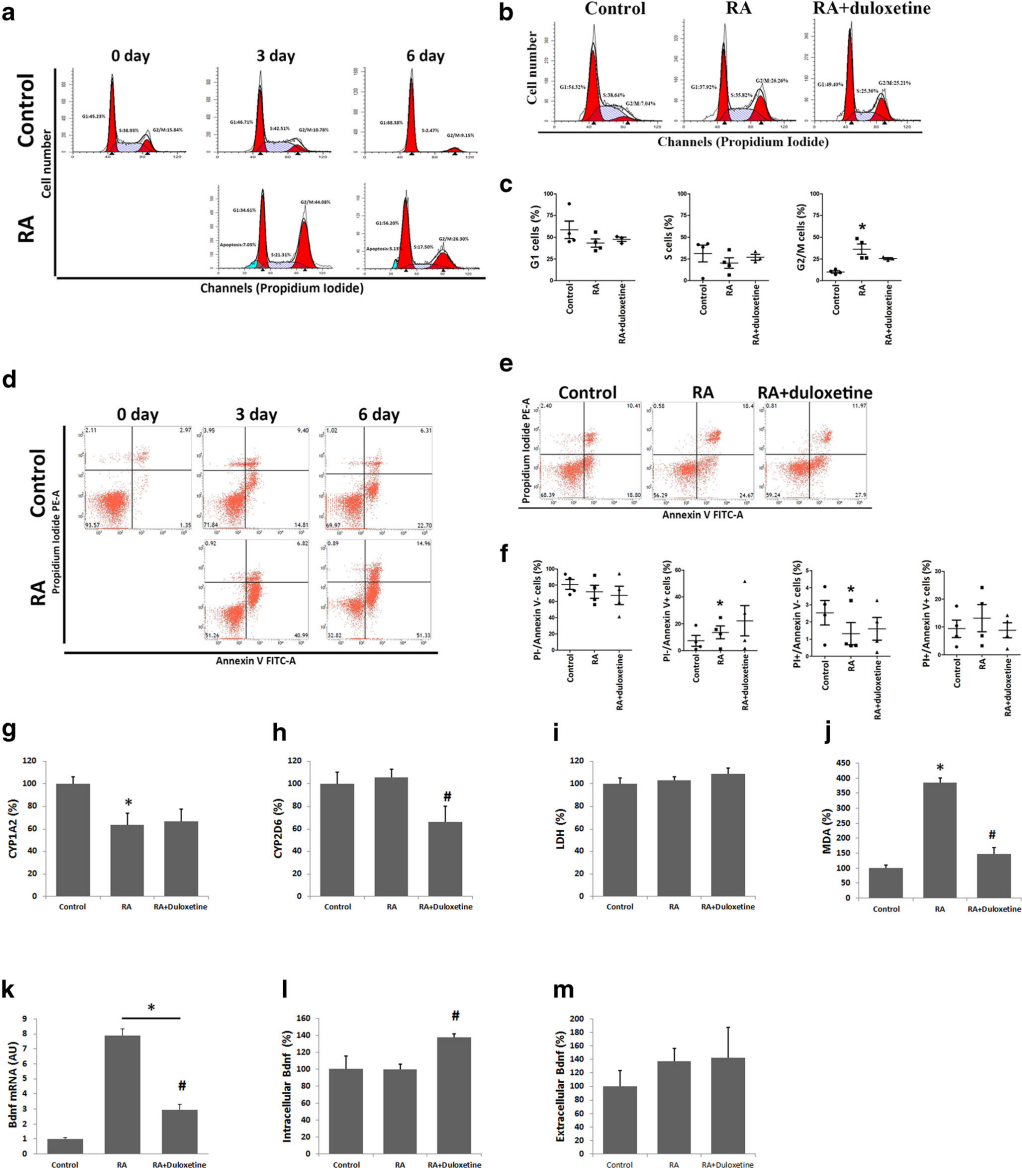
Duloxetine decreased CYP2D6 and MDA during the induction of neurite outgrowth in N2a cells. Cell cycle (**a**) and annexin V- and PI-positive cells (**d**) were recorded at various time points during the induction of neurite outgrowth in N2a cells. The cell cycle (**b** and **c**) and the percentages of annexin V- and PI-positive cells (**e** and **f**), protein levels of CYP1A2 (**g**) and CYP2D6 (**h**), MDA (**i**), LDH (**j**), mRNA(**k**) and protein of mouse Bdnf of intracellular (**l**) and extracellular (**m**) were assayed and analyzed statistically, after N2a cells were differentiated or differentiated in the presence of duloxetine (12.5 μM) for 24 h. (*n* = 3, *P < 0.05 versus control group, #*P* < 0.05 versus control group)

For cell cycle analysis, PI was used to determine the distribution of the DNA content. The populations of the cells in the S and G2/M phases were low in the control and RA-differentiated groups, and the RA-differentiated group showed an increased G2/M phase cell population compared with the normal culture group (Fig. 5a). There was a significant increase in the G2/M phase cells after 24 h of the differentiation treatment, but duloxetine did not increase this change (Fig. 5 b and c).

To evaluate cell death, PI and annexin V were used to label the dead cells. The proportions of the PI- and annexin V-positive cells were increased in the control cells and the cells subjected to the RA differentiation culture process, and the RA differentiation group showed an increase in the numbers of dead cells compared with the normal culture group (Fig. 5d). There was a significant increase in the PI-negative and annexin V-positive cells and a decrease in the PI-positive and annexin V-negative cells after the 24-h differentiation treatment, but duloxetine did not increase this change (Fig. 5 e and f).

With respect to the biochemical activities, the duloxetine-induced cell toxicity that was associated with CYP1A2, CYP2D6, and LDH activities in the supernatants, and MDA levels of cell lysate were assayed. There was a significant decrease in the CYP1A2 level in the RA-differentiated group compared with the control group, but duloxetine did not enhance this change (Fig. 5g). CYP2A6 did not change during the induced differentiation, but duloxetine caused a significant decrease (Fig. 5h). There was a change in the LDH in the RA and RA + duloxetine groups (Fig. 5i). RA differentiation increased the MDA level, whereas duloxetine decreased this change (Fig. 5j).

BDNF was a potential target of duloxetine pharmacological action (Mannari et al. 2008). Protein and mRNA levels of mouse Bdnf were assayed after N2a cells committed 24-h RA differentiation with or without duloxetine. RA differentiation induced Bdnf mRNA expression in N2a cells (Fig. 5k). Duloxetine decreased Bdnf mRNA expression (Fig. 5k), increased Bdnf protein level of intracellular (Fig. 5l), and did not affect suspension protein level (Fig. 5m) compared with the RA differentiation group without duloxetine.

## Discussion

The main psychotherapeutic effect of duloxetine is the upregulation of SER and NOR in the CNS by reuptake inhibition, and the toxicity of duloxetine was due to the inhibition of cytochrome P450 (CYP) enzyme level. The neural protection function of duloxetine has been confirmed in many cell types including rat primary cells (Demirdas et al. 2017; Hisaoka-Nakashima et al. 2016), immortalized cell lines (Akpinar et al. 2014; Stoetzer et al. 2016), and human cell lines (Engel et al. 2018). The doses of duloxetine in cell biology experiments differed from its physiological concentration. In clinical studies, after twice daily oral doses of 60 mg, the maximum plasma concentration of duloxetine in adults was 0.48 μM or 144 ng/ml (highest dose studied for efficacy) (Knadler et al. 2011). Duloxetine treatment at 20 μM induced human neuroblastoma SH-SY5Y cell death (Engel et al. 2018), whereas this agent showed some protective effect of against oxidative stress–induced cell death following treatment with 1–5 μM (for SH-SY5Y cells) (Engel et al. 2018) or 10 μM (rat pheochromocytoma PC12 or neurons) (Akpinar et al. 2014; Demirdas et al. 2017). In this study, we tested duloxetine’s neural cell toxicity in the mouse neuroblastoma cell line N2a and mouse C17.2 neural stem cells. These specific cells were good models for the evaluation of clinical drug toxicity (Wang et al. 2019). The results showed significant cell toxicity in both cell lines when they were exposed to 100 μM duloxetine, and the C17.2 cells were more sensitive than the N2a cells as shown by the induction of cell death by a lower dose. The duloxetine-induced neural cell death was not associated with changes in either extracellular or intracellular serotonin or norepinephrine concentrations. Duloxetine induced typical dose-dependent cell death phenomena in N2a cells, including changes in the cell viability, cell morphology, cell markers, and biochemistry as well as a reduction in the CYP level. These results suggest that duloxetine could be a potential agent to kill cancer cells if administered via local injection.

Duloxetine was already used for treating neural disease, but some additional testing should be completed before anti-cancer use by centrally administering the test. Duloxetine is a safe drug for oral administration. The physiological concentration of neural system by gastrointestinal absorption was not associated with significant pathology damages. For anti-cancer usage by centrally administering the treatment, firstly, the total dose should not be over orally administered; secondly, drug spread in solid neuroblastoma must be under control to make sure the drug and drug-induced DAMPs (damage-associated molecular patterns) go swiftly into the circulatory system without damaging other neural cell populations.

For anti-cancer drugs, the manner by which they cause cell death should be clear, and the cytotoxic effect should not be easily inhibited. The CNS is a complex system that includes many cell types and complicated cell signal communications. The microenvironment of the CNS affects which drugs are chosen to kill nerve tumors, because some forms of cell death are inhibited by neurotransmitters (Wang et al. 2016). To elucidate the manner of the cell death, we examined which inhibitor affected the changes in the viability of the N2a cells that were induced by 25 μM duloxetine. Trolox is an antioxidant, folic acid is essential for cell cycle process, rifampicin is a CYP inducer, rapamycin and chloroquine are an autophagy inducer and inhibitor, respectively, necrostatin-1 prevents necrosis, Z-VAD prevents apoptosis, and ferrostatin-1 prevents ferropotosis. Surprisingly, with the exception of rifampicin, most of the inhibitors did not affect the duloxetine-induced N2a cell death. Duloxetine and rifampicin could cause synergistic effects on N2a cell death by inhibiting CYP1A2 and CYP2D6 level. Rifampicin is an inducer of CYP1A2 (Backman et al. 2006) and CYP2D6 (Hellum et al. 2007) in individuals or hepatocytes. In the current study, we demonstrated that the duloxetine-induced cell death did not demonstrate typical cell death patterns as indicated by the limited effects of the inhibitors. Furthermore, rifampicin promoted the duloxetine-induced cell-killing effect by inhibiting the CYP level.

Although there were differences between N2a cells and primary cultured neurons (LePage et al. 2005), N2a cells are an approved neural differentiation model (Koike et al. 2015). In N2a cells, neural differentiation was induced by withdrawal of serum (Evangelopoulos et al. 2005) and the addition of all-trans retinoic acid (RA) (Marzinke and Clagett-Dame 2012) and growth factors. This differentiation process is characterized by cell proliferation attenuation and neurite outgrowth (Dasgupta and Milbrandt 2007). We found that duloxetine promoted the differentiation induced by serum withdrawal and RA addition in the N2a cells, and rifampicin was not effective. There were not any direct causal relationships between N2a neurite outgrowth and cell death as indicated by evidence that various factors could promote cell death and neurite outgrowth together (Kuenzi et al. 2008) or inhibit neurite outgrowth and promote cell death (Ye et al. 2008). Because cell cycle arrest is one of the potential mechanisms for cell death (Gire and Dulic 2015), cell cycle, cell death, and biochemical were assayed. We found that duloxetine did not promote the differentiation-induced cell cycle and cell death changes. The possible reasons by which duloxetine led to N2a neurite outgrowth were the inhibition of CYP2D6 levels, a reduction in the intracellular MDA levels, and an induction of Bdnf protein levels of intracellular and extracellular. There was a contradiction in the mRNA and protein expression in our study. The relationship of mRNA and protein level was complex, as positive and negative feedback regulation of the function of the gene both existed (Zhdanov 2018). As our assay was taken in 24 h of differentiation, the possible illustration could be the increase of Bdnf protein was a negative feedback regulation of mRNA level. Some further studies are needed to define the relationship between duloxetine-induced Bdnf mRNA and protein expression.

## Conclusion

In summary, we demonstrated that duloxetine induced neural cell death and promoted neurite outgrowth in N2a cells. The duloxetine-induced neural cell death was promoted by rifampicin but could not be reversed by classic cell death inhibitors. The N2a cell neurite outgrowth could be promoted by duloxetine via reduction of CYP2D6 and MDA levels and induction of Bdnf protein levels. Additional studies are needed to determine whether the pathway of duloxetine-induced neural cell death differs from the classic cell death pattern, whether duloxetine promotes nerve reconstruction in primary neurons and CNS damage models, and the relationship between duloxetine-induced Bdnf mRNA and protein expression.

## Data Availability

All data generated or analyzed during this study are included in this published article (and its supplementary information files).

## Abbreviations

CNS: Central nervous system
CYP: Cytochrome P450 proteins
Bdnf: Brain-derived neurotrophic factor
SNRIs: Serotonin–norepinephrine reuptake inhibitors
SER: Serotonin
NOR: Norepinephrine
CCK-8: Cell counting kit-8
FBS: Fetal bovine serum
PI: Propidium iodide
TUNEL: TdT-mediated dUTP nick-end labeling
RA: Retinoic acid
DMEM: Dulbecco’s modified eagle’s medium
ELISA: Enzyme-linked immunosorbent assays
LDH: Lactate dehydrogenase
MDA: Malondialdehyde
